# Up-regulation of dorsal root ganglia BDNF and trkB receptor in inflammatory pain: an *in vivo *and *in vitro *study

**DOI:** 10.1186/1742-2094-8-126

**Published:** 2011-09-30

**Authors:** Ya-Tin Lin, Long-Sun Ro, Hung-Li Wang, Jin-Chung Chen

**Affiliations:** 1 Department of Physiology and Pharmacology, Chang-Gung University, Taiwan, Republic of China; 2 Graduate Institute of Biomedical Sciences, Chang-Gung University, Taiwan, Republic of China; 3 Department of Neurology, Chang-Gung Memorial Hospital, Taiwan, Republic of China

## Abstract

**Background:**

During inflammation, immune cells accumulate in damaged areas and release pro-inflammatory cytokines and neurotrophins. Brain-derived neurotrophic factor (BDNF) plays a neuromodulatory role in spinal cord dorsal horn via the post-synaptic tyrosine protein kinase B (trkB) receptor to facilitate pain transmission. However, the precise role of BDNF and trkB receptor in the primary sensory neurons of dorsal root ganglia (DRG) during inflammation remains to be clarified. The aim of this study was to investigate whether and how BDNF-trkB signaling in the DRG is involved in the process of inflammatory pain.

**Methods:**

We used complete Freund's adjuvant- (CFA-) induced and tumor necrosis factor-α- (TNF-α-) induced inflammation in rat hindpaw as animal models of inflammatory pain. Quantification of protein and/or mRNA levels of pain mediators was performed in separate lumbar L3-L5 DRGs. The cellular mechanism of TNF-α-induced BDNF and/or trkB receptor expression was examined in primary DRG cultures collected from pooled L1-L6 DRGs. Calcitonin gene-related peptide (CGRP), BDNF and substance P release were also evaluated by enzyme immunoassay.

**Results:**

CFA injection into rat hindpaw resulted in mechanical hyperalgesia and significant increases in levels of TNF-α in the inflamed tissues, along with enhancement of BDNF and trkB receptor as well as the pain mediators CGRP and transient receptor potential vanilloid receptor subtype 1 (TRPV1) in DRG. Direct injection of TNF-α into rat hindpaw resulted in similar effects with retrograde transport of TNF-α along the saphenous nerve to DRG during CFA-induced inflammation. Primary DRG cultures chronically treated with TNF-α showed significant enhancement of mRNA and protein levels of BDNF and trkB receptor, BDNF release and trkB-induced phospho-ERK1/2 signal. Moreover, CGRP and substance P release were enhanced in DRG cultures after chronic TNF-α treatment or acute BDNF stimulation. In addition, we found that BDNF up-regulated trkB expression in DRG cultures.

**Conclusions:**

Based on our current experimental results, we conclude that inflammation and TNF-α up-regulate the BDNF-trkB system in DRG. This phenomenon suggests that up-regulation of BDNF in DRG may, in addition to its post-synaptic effect in spinal dorsal horn, act as an autocrine and/or paracrine signal to activate the pre-synaptic trkB receptor and regulate synaptic excitability in pain transmission, thereby contributing to the development of hyperalgesia.

## Background

Inflammation and pain largely share a common course of progression; patients with inflammation may suffer hyperalgesia and/or allodynia to various mechanical, thermal and chemical stimuli [1]. Inflammation results in an array of chemical mediators being released and triggering immune cell accumulation in the damaged area. Those activated immune cells further release pro-inflammatory cytokines and neurotrophins including nitric oxide (NO), interleukin-1β (IL-1β), interleukin-6 (IL-6), tumor necrosis factor-α (TNF-α) and nerve growth factor (NGF) [1-3] producing either central or peripheral sensitization [3-5].

TNF-α is a potent pro-inflammatory cytokine that has been used frequently in laboratory studies to evoke inflammatory reactions. TNF-α activates the release of many cytokines, such as IL-1β, IL-6 and IL-8, and participates in the development of inflammatory hyperalgesia mainly through its receptor, TNFR1 and TNFR2 [6-8]. TNF-α-dependent neuropathy or inflammatory pain appears to be largely mediated by TNFR1 [9-11].

Neurotrophins like NGF, neurotrophin 3/4 (NT-3/4) and brain-derived neurotrophic factor (BDNF) can be released from DRG, acting to either support neuronal development [12] or participate in the induction of hyperalgesia [3]. NGF is recognized to play a potent role in the development of neurogenic pain by inducing hyperalgesia [5,13]. After release from immune cells, NGF up-regulates the expression of proteins involved in inflammatory pain transmission, TRPV1, BDNF, calcitonin gene-related peptide (CGRP) and substance P in the DRG via tyrosine protein kinase A (trkA) receptor [2,3,14-17]. BDNF is expressed and synthesized in small- to medium-sized DRG neurons and co-expressed with trkA along with CGRP and substance P [18,19]. Hence, BDNF can be released in response to peripheral NGF via trkA stimulation and is known as a nociceptive modulator for both pain perception and sensitization at both spinal and supraspinal levels [18]. In particular, nociceptor-drived BDNF has been demonstrated to regulate acute and inflammatory pain [20]. Tyrosine protein kinase B (trkB) is a high affinity BDNF receptor [18]. Recent ultrastructural evidence indicates that trkB receptor is not only expressed in post-synaptic neurons but also localizes to pre-synaptic terminals in spinal lamina II [21]. BDNF in spinal cord lamina II is a neuromodulator of nociception at synapses. Recent studies indicate that BDNF acts on pre-synaptic trkB receptors to increase the frequency of glutamatergic EPSCs in spinal dorsal horn of complete Freund's adjuvant- (CFA-) treated rats [22,23]. Hence, BDNF-induced thermal hyperalgesia can be inhibited by intrathecally applied NMDA receptor antagonist D-APV [24]. Further, it has been found that trkB phosphorylation is increased under noxious mechanical, thermal or chemical stimulation [25]. This effect is tightly associated with extracellular signal-regulated kinase (ERK) and phospholipase C (PLC)/phosphoinositide-3 kinase (PKC) signals [18].

The aim of this study was to investigate the role of BDNF-trkB within DRG during the progression of inflammatory pain, specifically focusing on whether and how the pro-inflammatory cytokine TNF-α influences trkB expression and signaling. To accomplish this aim, we used CFA-induced inflammation in rats as a chronic pain model to examine neurochemical changes in lumbar DRG. Levels of the pro-inflammatory cytokine TNF-α were first determined in the inflamed tissues. Consequently, a set of animals was injected with TNF-α directly into hindpaw to assess inflammatory pain responses. In addition, we used DRG primary cultures and treated with TNF-α for mechanistic and functional studies. We demonstrate that BDNF and trkB receptors are up-regulated in DRG of CFA- and TNF-α-induced inflammatory pain models. In addition, retrograde transport of TNF-α was observed in the CFA-induced inflammation model. Both trkB receptors and trkB-induced ERK signaling were increased after chronic TNF-α-treatment in DRG primary cultures. Furthermore, both CGRP and substance P release were enhanced after chronic TNF-α pre-treatment or acute BDNF administration in DRG cultures. We conclude that experimental arthritis and TNF-α initiate a BDNF-trkB feed-forward pathway in peripheral sensory neurons, and this up-regulation of BDNF-trkB system may participate in pain sensitization.

## Methods

### Animals

Male Sprague-Dawley (SD) rats (BioLASCo Taiwan Co., Ltd) weighing 250-280 g (for inflammatory animal model) or 3-4 weeks of age (for primary culture) were used in the current study. After arrival, animals were acclimatized to the room with controlled temperature (22 ± 2°C), air humidity (50 ± 10%) and 12 hours day-night cycle (light on at 7:00 AM) for at least 7 days before experimentation. The animals were housed 2 or 3 per cage with food (Western Lab 7001, Orange, CA, USA) and water *ad libitum*. The animal handling and drug treatments were performed in strict accordance with the NIH Guide for the Care and Use of Laboratory Animals, and all procedures were approved by the Animal Care Committee of Chang-Gung University.

### Chemicals

CFA (Sigma, St. Louis, MO, USA) was freshly prepared (1:1 with saline containing 0.05 mg heat-killed and dried Mycobacterium tuberculosis and paraffin oil mixed in a water-in-oil emulsion). Recombinant rat TNF-α protein (R & D systems, Minneapolis, MN, USA) was reconstituted with phosphate buffered saline (PBS)/0.1% bovine serum albumin (BSA). Recombinant human BDNF protein (Abcam, Cambridge, MA, USA) was reconstituted with ddH_2_O. NGF 2.5S (Chemican, Temecula, CA, USA) was reconstituted with PBS.

### Animal model of inflammatory pain

To produce an *in vivo *model of inflammatory pain, animals were anesthetized with isoflurane and locally injected with CFA, TNF-α or vehicle. In the CFA-treated group, animals were injected with 100 μl CFA into the right hindpaw with the contralateral side serving as an untreated control. Other animals received saline injection in the hindpaw to serve as a vehicle control. In the TNF-α-treated group, animals received 500 ng TNF-α (30 μl) in the hindpaw twice daily for three consecutive days, followed by daily mechanical *von Frey *tests. Lumbar L3~L5 DRGs or hindpaw tissues were collected 72 hours after CFA or TNF-α injection and samples of DRG were divided into following different groups: CFA (ipsilateral), TNF-α (ipsilateral), control (contralateral) and saline groups.

### Pain threshold test

Mechanical hyperalgesia was measured by the *von Frey *method [26]. The test was performed daily in CFA- or TNF-α-treated rats for a period of up to three days. To perform the von Frey test, rats were placed in a clear plexiglass box (20 × 19 × 20 cm) on an elevated mesh screen. A calibrated von Frey rigid tip (Electronic von Frey Anesthesiometer, IITC Life Science, USA) was applied to the plantar surface of each hindpaw in a series of logarithmically ascending forces. The responses were recorded in grams of paw withdrawal averaged over three to five applications referred to as mechanical pain threshold. The interval of each application was 5 min.

### Primary DRG culture

Bilateral lumbar L1-L6 DRGs were dissected from 3-4-week-old SD rats. DRG tissues were digested with 1 mg/ml collagenase (Sigma) at 37°C for 30 min and then incubated with 0.25% (v/v) trypsin-EDTA (Biological Industries, Israel) at 37°C for 30 min. Tissues were centrifuged at 300 × *g *for 5 min and then re-suspended in DMEM/F12 medium (repeated three times). Next, tissues were manually triturated approximately sixty times using a flame-polished Pasteur pipette. The dissociated cells were suspended in DMEM/F12 medium containing 10% fetal calf serum, 100 μg/ml penicillin/streptomycin and 1 mM sodium pyruvate and plated onto poly-L-lysine-coated plates. Medium was replaced the following day with the addition of 10 μM Ara-C and 100 ng/ml NGF and changed every two days thereafter. After cell plating for two days, all drugs (TNF-α, BDNF or NGF) were added for the designated time periods and samples (medium or cultured cells) were all collected by the end of each experiment (including basal control).

### Western blot

All samples were sonicated in 1% (w/v) sodium dodecyl sulfate (SDS) and heated to 100°C for 5 min. Protein concentration was determined by the Coomassie blue method with bovine serum albumin as standard. Equal amounts of proteins (10-40 μg) were separated by 10% SDS-polyacrylamide gel electrophoresis and transferred onto a polyvinylidene difluoride (PVDF) membrane (Millipore Corporation, Bedford, MA, USA) using 200 mA for 70 min at 4°C. Immunoblots were first blocked with 5% (w/v) non-fat milk for 1 hour, followed by incubation overnight with specific primary antibody. The antibodies used were anti-β-actin antibody (1:1000; Sigma), anti-phospho-p44/42 MAP kinase (Thr202/Tyr204) antibody (anti-phospho-ERK1/2, 1:1000; Cell Signaling, Beverly, MA, USA). On the second day, blots were incubated with secondary antibody conjugated with HRP (1:2000-1:10,000, GE Healthcare, UK) for 1 hour. After another 3 × 10 min washes with TBS-T buffer, the substrate solution (Amersham ECL kit) was applied for 1 min. The enhanced chemiluminescence signal was detected with X-ray film (Kodak, X-OMAT) and quantified by laser scanning densitometry (Molecular Dynamics, Sunnyvale, CA). Signals were normalized to β-actin.

### Quantitative real-time PCR

Total RNA was isolated from cultured DRG neurons or tissues using TRIzol^® ^reagent (Invitrogen, Carlsbad, CA, USA) according to the manufacturer's protocol. The mRNA was transcribed to cDNA via reverse transcriptase (HT BioTechnology, England UK). The cDNA for corresponding targets was measured by quantitative real-time PCR using a PE Applied Biosystems prism model 7000 sequence detection instrument (Applied Biosystems, Foster City, CA, USA). The PCR program was: 95°C for 10 min then 95°C for 15 sec and 60°C 30 sec for 40 cycles. SYBR (Invitrogen) was used as fluorescent dye and ROX as reference dye. The primer sequences are as follows: 18S-forward, 5' GCT GTG GTC CAA GGC CAT TTT 3'; 18S-reverse, 5' CCG AGT TAC TTT TCC CCA GAT GAC 3'[GenBank: NM_000996]; TRPV1-forward, 5' GCG AGT TCA AAG ACC CAG AG 3'; TRPV1-reverse, 5' GGC ATT GAC AAA CTG CTT CA 3'[GenBank: NM_031982]; trkB.FL-forward, 5' AAG ATC CTG GTG GCC GTG AAG A 3'; trkB.FL-reverse, 5' CGG CTT CGC GAT GAA AGT CCT T 3'[GenBank: NM_012731]; BDNF-forward-1, 5' CAA GGC AAC TTG GCC TAC CC 3'; BDNF-reverse-1, 5' GAG CAT CAC CCG GGA AGT GT 3'; BDNF-forward-2, 5' TTA CCT GGA TGC CGC AAA CA 3'; BDNF-reverse-2, 5' TGG CCT TTT GAT ACC GGG AC 3' [GenBank: NM_012513]; CGRP-forward, 5' AAC CTT AGA AAG CAG CCC AGG CAT G 3'; CGRP-reverse, 5' GTG GGC ACA AAG TTG TCC TTC ACC A 3'[GenBank: M11597]. Threshold cycle, Ct, which correlates inversely with the target mRNA levels, was measured. ABI Prism Primer Express was used to design the specific gene primers. After normalization to 18S values, data were expressed as percentage of corresponding controls.

### Immunohistochemistry (IHC) for tissue sections

SD rats were sacrificed by decapitation; L3-L5 DRGs were removed and post-fixed in 4% (w/v) paraformaldehyde solution for 48 hours at 4°C, then cryo-protected with 20% (w/v) sucrose in potassium phosphate buffer saline (KPBS, KH_2_PO_4 _3.3 mM, K_2_HPO_4 _21.9 mM, NaCl 154 mM) overnight at 4°C. IHC images were collected from 14 μm sections prepared using a freezing microtome (LEICA CM3050S, Bannockburn, IL, USA).

Tissue sections were rinsed twice with KPBS. Endogenous peroxidase was quenched by pre-treating the sections with 0.03% (v/v) H_2_O_2_. The sections were then rinsed with KPBS followed by pretreatment with 1% (w/v) sodium tetrahydridoborate for 5 min. Afterward, the sections were incubated in primary antibody in 2% (w/v) normal goat serum/0.3% (v/v) TX-100/KPBS at 4°C for 48 hours. The antibodies used were anti-trkB antibody (1:2000; Santa Cruz, Santa Cruz, CA, USA), anti-BDNF antibody (1:1000; Abcam), anti-CGRP antibody (1:8000; Chemicon). Afterward, sections were incubated with 1:200 biotinylated goat anti-rabbit IgG secondary antibody for 1 hour. Immunoreactivity was visualized by the avidin-biotin complex method (Vector Lab., Burlingame, CA, USA). After incubating with the avidin-biotin complex for 1 hour, sections were developed using 3, 3'-diaminobenzideine (DAB; Sigma) and nickel ammonium sulfate in KPBS. Sections were mounted onto gelatin-coated slides, dehydrated through a serial alcohol gradient, degreased with xylene and then covered slipped with Entellan (Merck, Germany). Co-localization of BDNF and TNFR1 was evaluated by immunofluorescent double stain. Primary antibodies (anti-BDNF, 1:200; Abcam and anti-TNFR1, 1:20; Santa Cruz) were mixed and incubated at 4°C for 72 hours. After washed with KPBS, sections were incubated with secondary antibodies (Rodamine-conjugated goat-anti-mouse and Dylight 488-conjugated goat-anti-rabbit, 1:200, Jackson ImmunoResearch, West Grove, PA, USA) for 1 hour at room temperature. Sections were then incubated with 1:1000 DAPI (4',6-diamidino-2-phenylindole, Roche, Switzerland) for 5 min before mounting with glycerol. Signals of immunofluorescent stain were observed by fluorescence microscope.

### Immunocytochemistry for cultured cells

Cultured DRG neurons were fixed with 2% (w/v) paraformaldehyde solution (2% paraformaldehyde containing 0.3% TX-100, pH 7.2) for 30 min. Cells were rinsed with PBS/0.3% TX-100 following blocking with 2% normal goat serum in PBS/0.3% TX-100 for 30 min. Cells were then incubated in primary antibody in PBS/0.3% TX-100/2% goat serum at 4°C for 48 hours. The antibodies used were anti-trkB antibody (1:2000), anti-BDNF antibody (1:1000), anti-CGRP antibody (1:8000). TrkB staining was visualized via immunofluorescence. After rinsing three times, cells were incubated with FITC-conjugated goat anti-rabbit IgG secondary antibody (1:200, Jackson ImmunoResearch) for 1 hour. Afterward, cells were then incubated with 1:1000 DAPI for 5 min before mounting with glycerol. Co-localization of TNFR1 and phospho-ERK were evaluated by immunofluorescent double stain. Primary antibodies (anti-TNFR1, 1:20 and anti-phospho-ERK1/2, 1:250) were mixed and incubated at 4°C for 72 hours. After wash with PBS, secondary antibodies, Rodamine-conjugated goat anti-mouse and Dylight 488-conjugated goat anti-rabbit (1:200, Jackson ImmunoResearch), were also mixed together and added for 1 hour. Slides were then incubated with 1:1000 DAPI for 5 min before mounting with glycerol. Immunofluorescent stain signal was observed by fluorescence microscope. On the other hand, BDNF and CGRP staining were visualized via avidin-biotin complex method. Similar to immunohistochemistry, endogenous peroxidase was quenched by pre-treating the sections with 0.03% H_2_O_2 _followed by pretreatment with 1% sodium tetrahydridoborate. After incubating with primary antibody and rinsing with PBS/0.3% TX-100, the cells were incubated in 1:200 biotinylated goat anti-rabbit IgG secondary antibody for 1 hour and rinsed with PBS. After incubation with avidin-biotin complex for 1 hour, ABC reaction was performed with a mixture of DAB and nickel ammonium sulfate. Cells were mounted on slides, dehydrated, and cover-slipped with mounting solution. The color of precipitate formed by DAB was monitored microscopically.

### Enzyme immunoassay (EIA)

The release of BDNF, CGRP and substance P were determined using BDNF E_max_^® ^ImmunoAssay system (Promega, Madison, WI, USA), CGRP EIA kit (SPIbio, France) or substance P EIA kit (Cayman, Ann Arbor, Michigan, USA), respectively. For BDNF assay, after 5 nM TNF-α treatment for 48 hours, medium of DRG cultures were collected. The cells were lysised, sonicated and then centrifuged at 10,000 × *g *for 15 min. The supernatants were analyzed and represented as total BDNF content. For CGRP and substance P EIA assay, after 5 nM TNF-α treatment for 48 hours, culture medium were replaced by a fresh medium and allowed cells stable for 2 hours. Afterwards, 400 ng/ml BDNF was added for 30 or 60 min and the supernatant was collected and analyzed immediately according to the manufacturer's protocols. The microplate was read at 405 nm (yellow color) in an enzyme-linked immunosorbent assay (ELISA) plate reader (ASAY microplate reader, expert 96, Austria).

The amount of TNF-α in rat hindpaw or DRG was evaluated using a rat TNF-α EIA kit (R&D system). After CFA-injection for 72 hours, rat hindpaw or L3~L5 DRGs were isolated and analyzed immediately. Tissues were collected in PBS solution containing proteinase inhibitors leupeptin, aprotenin and PMSF (Sigma). After homogenization, tissues were centrifuged at 10,000 × *g*, 4°C for 20 min and supernatants were collected. Blood samples were withdrawn from the animals' trunks into a blood collection tube containing 5.4 mg K2 EDTA and centrifuged at 1,000 × *g *for 10 min in the room temperature to collect the serum. Samples were analyzed immediately according to the manufacturer's protocols. The concentration of TNF-α was determined by the absorbance at 450 nm with reference filter at 540 nm.

### TNF-α retrograde transport tracing

Recombinant rat TNF-α protein (10 μg) was labeled with Alexa Fluor 488 (Molecular Probes, Invitrogen) according to the manufacturer's protocols. Animal received Alexa Fluor 488-TNF-α (25 μl) in CFA pre-treated hindpaw 24 hours before sacrifice (72 hours after CFA injection). Alexa Fluor 488 (25 μl) injection in CFA pre-treated hindpaw served as control. After injection, ipsilateral saphenous nerve was isolated immediately and monitored under fluorescence microscope. The fluorescent signals in the saphenous nerve were separately measured in three segments: distal (0.5 cm from the heel), middle (1.3 cm from the heel) and proximal (2 cm from the heel).

### TUNEL reaction

Cell apoptosis was evaluated using an *in situ *cell death detection kit (TUNEL reaction, Roche, Germany), following the manufacturer's protocols, DRG primary cultures were fixed with 2% paraformaldehyde for 1 hour, then incubated with 0.1% Triton X-100/0.1% sodium citrate for 2 min on ice. Afterwards, the cultures were washed with PBS and then treated with 50 μl TUNEL reaction mixture (containing green Fluorescein) at 37°C for 60 min in the dark. After enzyme reaction, cultures were washed with PBS and double stained with DAPI (1:1,000) for 5 min. Numbers of apoptotic cells were evaluated by observation under a fluorescence microscope.

### Data analyses and statistics

ImageJ (NIH, Bethesda, MD, USA) software was used to quantify the immunostaining images. A threshold was set by ImageJ to define the positive neurons among different experiments of the same study. For DRG immunostaining, three different sections (spacing 250~300 μm) were selected to calculate the total number of positive neurons and expressed as the percentage of positive neurons relative to the total number of neurons visualized in DRG sections. For the data in Table 1, small, medium and large DRG neurons were identified and counted based on the diameter of the neuron: small neurons < 25 μm; medium neurons 25 to 40 μm; and large neurons > 40 μm. The results were calculated by two methods: 1) percentage of trkB-positive neurons in specific categories (small, medium, or large) relative to the total number of neurons in DRG sections; 2) percentage of trkB-positive neurons in specific categories relative to the total number of trkB-positive neurons in the DRG sections.

All data were analyzed with GraphPad InStat (GraphPad Software, San Diego, CA, USA). Results are expressed as mean ± SEM. The data were analyzed by one-way ANOVA followed by Tukey multiple comparisons test or un-paired Student's t-test. A value of *p *< 0.05 was considered significant.

## Results

### Establishment of a CFA-induced inflammation animal model

**Figure 1:**
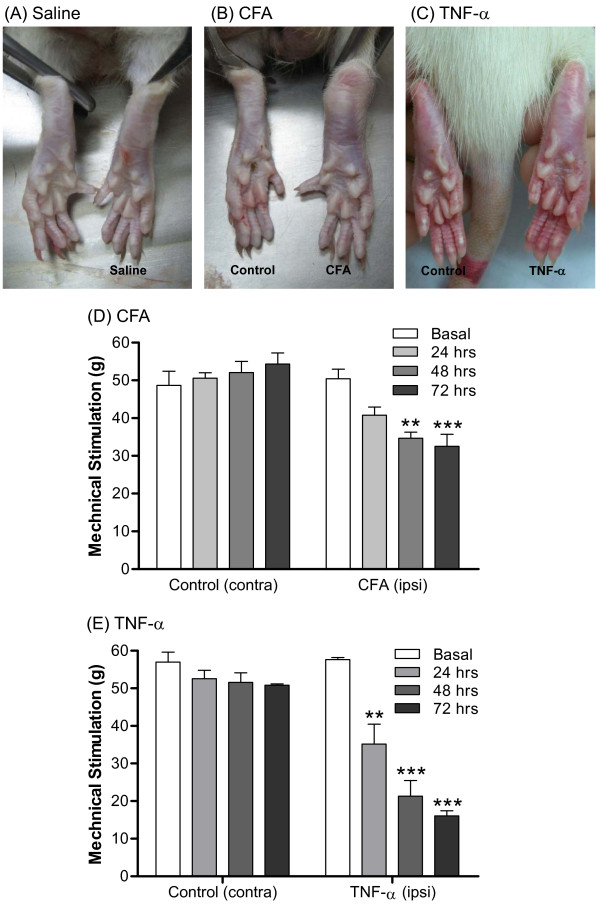
**Appearance of the rat hindpaw and *von Frey *hyperalgesia test after intraplantar CFA or TNF-α injection**. Seventy-two hours after injection of CFA, TNF-α or saline (control) into the right hindpaw of SD rats, we observed the hindpaw (A-C). Daily hyperalgesia testing was carried out for three days (D, E). Saline: 0.9% NaCl (ipsilateral) injection. Control (contra): contralateral to CFA or TNF-α. CFA (ipsi): CFA (100 μl) injected. TNF-α (ipsi): 500 ng TNF-α (30 μl) injected. Data were analyzed using one-way ANOVA followed by Tukey test and are expressed as mean ± SEM. **p < 0.01, ***p < 0.001; compared to basal (SD rats, N = 5 per group for CFA injection and N = 3 per group for TNF-α injection).

SD rats receiving 100 μl CFA in the intraplantar surface developed mono-arthritis within 72 hours. CFA-injected hindpaw appeared swollen and enlarged by the third day after injection (Figure 1B). In contrast, the non-injected contralateral hindpaw and saline injected controls displayed no significant size change (Figure 1A).

To measure pain threshold, mechanical hyperalgesia was assessed in three experimental groups via daily *von Frey *testing. As shown in Figure 1D, the threshold of mechanical stimulation progressively decreased with time after CFA injection. Statistically significant differences compared to basal level were found on the second and third days. No inflammatory signs were observed in the saline-injected group within 72 hours (data not shown). This result is similar to those of the non-injected contralateral hindpaw.

**Figure 2:**
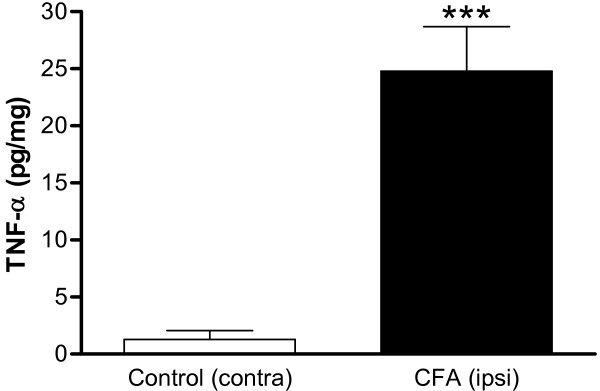
**Levels of TNF-α in hindpaw after intraplantar CFA injection**. Seventy-two hours after CFA injection, tissues from both hindpaw were collected and analyzed immediately by TNF-α EIA kit. Control (contra): contralateral to CFA, non-injected. CFA (ipsi): injected. Data are expressed as mean ± SEM and were analyzed using an un-paired Student's *t*-test. ***p < 0.001; compared to control (SD rats, N = 7 per group).

To determine if the pro-inflammatory cytokine TNF-α was involved in CFA-induced hyperalgesia, tissue content of TNF-α was measured in rat hindpaw, DRG and serum 72 hours after CFA-injection by a TNF-α EIA kit. As shown in Figure 2, TNF-α levels increased significantly in CFA-injected hindpaw as compared with contralateral hindpaw controls (1.28 pg/mg for control vs. 24.78 pg/mg for TNF-α-treated group). Levels of TNF-α in L3-L5 DRGs and serum did not reach the EIA detection limit (data not shown).

Further, to test whether TNF-α is involved in the development of inflammatory hyperalgesia, TNF-α was injected directly into rat hindpaw twice daily over a continuous three day period with mechanical hyperalgesia measured via *von Frey*. Similar to the CFA-injected animals, the hindpaw receiving TNF-α injection exhibited swelling (Figure 1C) accompanied by a time-dependent decrease in mechanical pain threshold (Figure 1E).

### Expression of inflammation- and pain-related proteins in DRG after CFA injection

**Figure 3:**
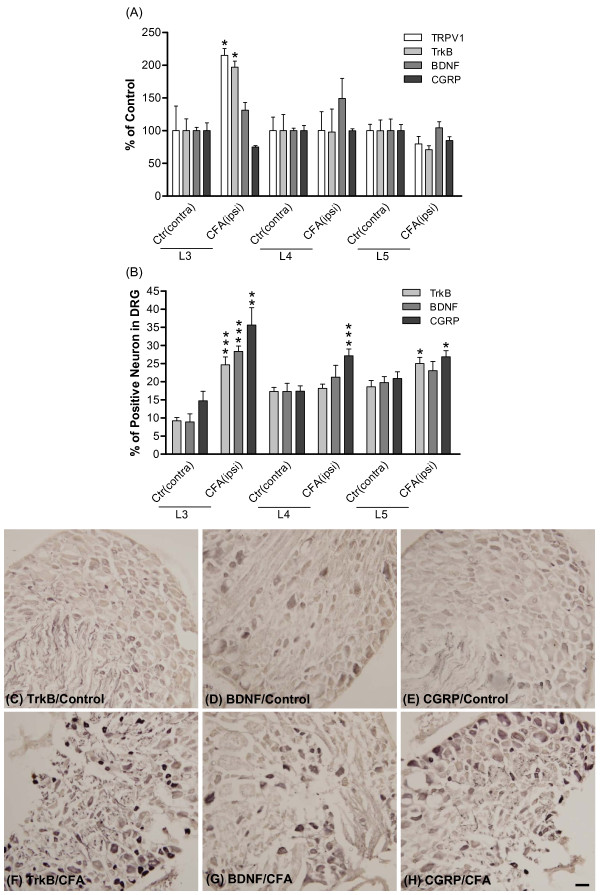
**Expression of TRPV1, trkB, BDNF and CGRP in DRG after intraplantar CFA injection**. Seventy-two hours after CFA injection, mRNA and protein levels of TRPV1, trkB, BDNF and CGRP were measured in L3-L5 DRGs. (A) mRNA levels were measured by real-time PCR. Values were normalized to 18S values, and are presented as percent of corresponding controls. Data are expressed as mean ± SEM and were analyzed by un-paired Student's *t*-test. *p < 0.05; compared to corresponding control groups (N = 3~6 per group, pooled from a total of 12 rats). (B) Quantitative measurements of trkB, BDNF and CGRP immunoreactivity in DRG sections. Protein levels were detected by the standard immunohistochemical ABC method. Data are presented as percentage of immuno-positive neurons in L3-L5 DRGs. (C-H) Photomicrographs illustrate the immunoreactivity of trkB, BDNF and CGRP in L3 DRG sections. (C-E), control group; (F-H), CFA group. Scale bars, 50 μm. Data are expressed as mean ± SEM and were analyzed using an un-paired Student's *t*-test. *p < 0.05, **p < 0.01, ***p < 0.001; compared to corresponding controls (contralateral to CFA injection). N = 9 per group. Control, ctr (contra): contralateral to CFA, non-injected. CFA (ipsi): injected.

To identify the underlying biochemical changes, rats were sacrificed 72 hours after CFA injection and L3-L5 DRGs were individually isolated. The mRNA levels for TRPV1, trkB, BDNF and CGRP were measured using real-time PCR. 18S ribosome RNA was used as internal control (Figure 3A). mRNA levels for TRPV1 and trkB were significantly increased in L3 DRG after CFA injection. On the other hand, mRNA levels for TRPV1, trkB and BDNF in L4 and L5 DRG after CFA injection showed no change compared to contralateral controls. To confirm this result, a second set of BDNF primers was used to analyze the tissues; however the results remained the same, i.e. no significant difference between control and CFA-treated groups in L3-L5 DRGs (data not shown). Further, CGRP mRNA expression did not change in L3-L5 DRG segments after CFA treatment.

Protein levels of BDNF, trkB and CGRP were also monitored via IHC detection in L3, L4 and L5 DRGs after CFA injection for 72 hours (Figure 3B-H). Protein expression was evaluated by counting numbers of positive neuron in DRG sections (Figure 3B). The results showed that the number of trkB receptor-positive neurons was significantly increased in both L3 and L5 DRGs, while the number of CGRP-positive neurons was increased significantly in all L3-L5 DRGs after CFA injection. Levels of BDNF protein increased significantly only in L3. Quantitative measurements of immunoreactivity in L3 sections showed that after CFA injection, the numbers of BDNF-, trkB- and CGRP-positive neuron were enhanced 3.18, 2.68 and 2.42 fold, respectively (Figure 3B).

To explore whether CFA-induced trkB over-expression occurs in a particular type of DRG neuron, trkB-positive neurons were separately counted based on the diameter of the neuron (small, medium and large neurons) in L3 DRG. As shown in Table 1, the percentage of trkB-positive neurons relative to total neurons in DRG was increased in small (*p *< 0.0001) and medium (*p *= 0.0025) neurons after CFA injection, with the percent increase of trkB-positive neurons equally distributed in three different size neuronal populations. It was noted that trkB receptors were mainly expressed in small-sized neurons (77.66% of control and 76.84% of CFA treated), moderately expressed in medium-sized neurons (16.42% of control and 17.35% of CFA treated) but rarely expressed in large-sized (5.92% of control and 5.81% of CFA treated) DRG neurons.

**Table 1:** Quantitative measurements of trkB-positive neurons in L3 DRG after CFA injection

	**Small**	**Medium**	**Large**
**Control**			
% to total neurons	10.19 ± 1.27	2.15 ± 0.38	0.85 ± 0.33
% to total trkB^**+ **^neurons	77.66 ± 3.15	16.42 ± 2.17	5.92 ± 2.34
**CFA**			
% to total neurons	20.7 ± 1.31***	4.94 ± 0.68**	1.63 ± 0.25
% to total trkB^**+ **^neurons	76.84 ± 19.3	17.35 ± 1.42	5.81 ± 0.71

Data expressed as mean ± SEM and analyzed using an un-paired Student's *t-*test, **p < 0.01, ***p < 0.001; compared to corresponding control (N = 9 per group).

**Figure 4:**
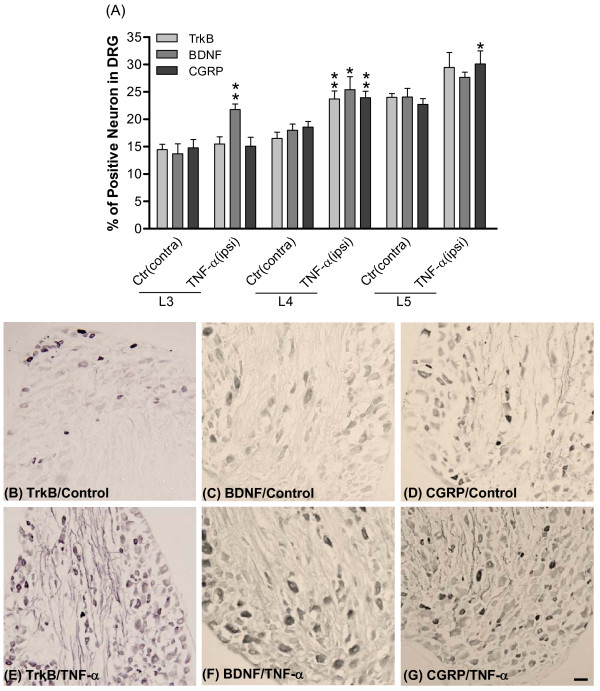
**Amount of trkB, BDNF and CGRP protein in DRG after intraplantar TNF-α injection**. After TNF-α injections (twice daily for three days), levels of trkB, BDNF and CGRP were measured in L3-L5 DRGs. Protein expression was detected by the standard immunohistochemical ABC method. (A) Quantitative measurements of trkB, BDNF and CGRP immunoreactivity in DRG sections. Data are presented as percentage of immuno-positive neurons in L3-L5 DRGs. (B-G) Photomicrographs illustrate the immunoreactivity of trkB, BDNF and CGRP in L4 DRG sections. (B-D), control group; (E-G), TNF-α group. Scale bars, 50 μm. Control, ctr (contra): contralateral to TNF-α. TNF-α (ipsi): injected. Data are expressed as mean ± SEM and were analyzed using an un-paired Student's *t*-test. *p < 0.05, **p < 0.01; compared to corresponding controls (contralateral to TNF-α injection). N = 9 per group.

The changes in expression of BDNF, trkB and CGRP protein in L3-L5 DRGs were also evaluated 72 hours after TNF-α injection (twice daily for three days) in the hindpaw. Data are presented as percentage of positive neurons in DRG. L3-L5 DRGs were counted separately. As shown in Figure 4, the amounts of BDNF, trkB and CGRP protein were significantly increased in L4 DRG. Similarly, the amount of BDNF and CGRP protein was enhanced in L3 and L5 DRGs, respectively. We found that the elevation of either protein after TNF-α treatment was less than that induced by CFA treatment (compared from Figure 4A, L4 and Figure 3B, L3).

**Figure 5:**
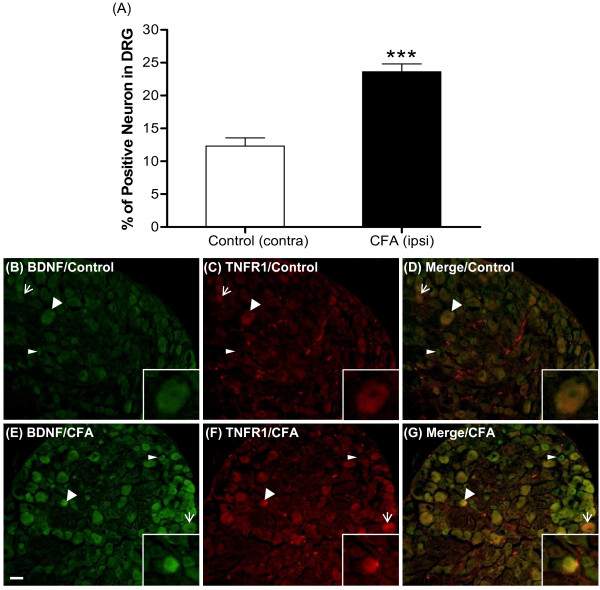
**Co-localization of BDNF and TNFR1 in the L3-L5 DRGs**. Immunoreactivity was detected by double immunofluorescent stain. L3-L5 DRGs were collected after CFA injection for 72 hours. BDNF is shown by green fluorescence and TNFR1 is shown by red fluorescence. (A) Bar graph representing quantitative measurement of numbers of cells displaying TNFR1 and BDNF co-localization. Data are expressed as mean ± SEM and were analyzed using an un-paired Student's *t-*test. ***p < 0.001 (N = 9 per group); compared to control. (B-D) Photomicrographs illustrate the immunoreactivity of BDNF (B) and TNFR1 (C) in the L3 DRG sections of control group. (D) Merged image of (B) and (C). (E-G) Photomicrographs illustrate the immunoreactivity of BDNF (E) and TNFR1 (F) in L3 DRG sections of the CFA group. (G) Merged image of (E) and (F). Inserts illustrate BDNF and TNFR1 signals that co-localized in a DRG neuron. Wide arrow, co-localized BDNF and TNFR1; narrow arrow, TNFR1; arrowhead, BDNF. Scale bars, 50 μm.

Previously, it was reported that under peripheral inflammatory conditions, TNF-α binds to TNFR1 and activates inflammatory signals by retrograde transport to the DRG [27]. Double fluorescent staining was thus used to test whether BDNF and TNFR1 were co-localized in L3-L5 DRGs 72 hours after CFA injection. As shown in Figure 5, BDNF protein was largely co-localized with TNFR1 and the number of co-localized TNFR1 and BDNF cells was significantly enhanced after CFA injection (Figure 5A). We also found that TNFR1 protein is largely localized in the cytoplasm (Figure 5B~G).

### Up-regulation of BDNF-trkB in cultured DRG neurons after TNF-α treatment

**Figure 6:**
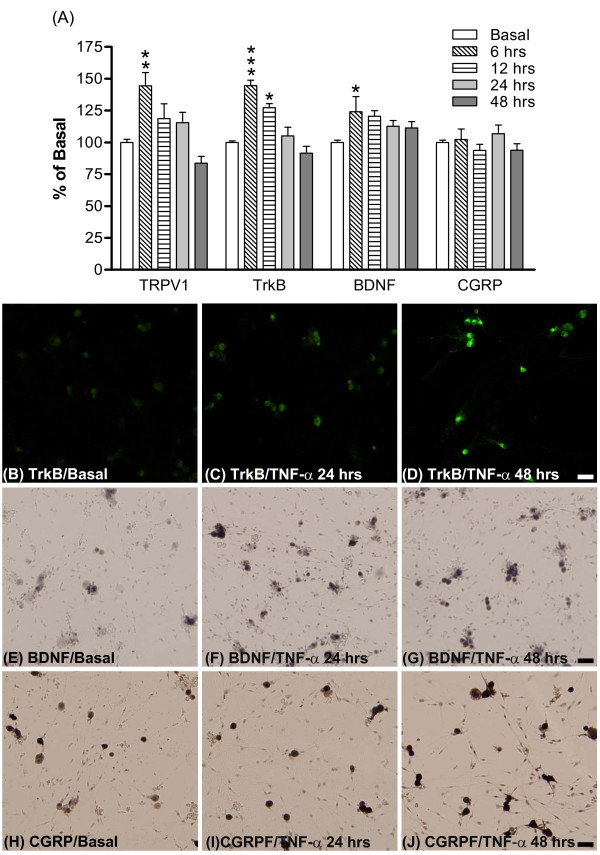
**Amount of TRPV1, trkB, BDNF and CGRP expression in DRG cultures after TNF-α treatment**. (A) Levels of TRPV1, trkB, BDNF and CGRP mRNA in DRG cultures (N = 3-7 per group). mRNA expression levels were assessed by real-time PCR after a 5 nM TNF-α treatment for 6 to 48 hours, and the results were normalized to 18S values. Data are expressed as mean ± SEM, presented as percentage of basal (no drug treatment) and were analyzed using one-way ANOVA followed by Tukey test. *p < 0.05, **p < 0.01, ***p < 0.001; compared to corresponding basal groups. (B-J) Photomicrographs illustrate the immunoreactivity of BDNF, trkB and CGRP in DRG cultures. The amount of protein was detected by immunofluorescent stain or the immunohistochemical ABC method after a 5 nM TNF-α treatment for either 24 or 48 hours in DRG cultures. (B, E, H) Basal protein expression (no drug treatment). (C, F, I) TNF-α treatment for 24 hours. (D, G, J) TNF-α treatment for 48 hours. Scale bars, 50 μm.

In order to investigate the cellular mechanism of inflammatory responses, primary DRG cultures were used (collected from bilateral L1~L6 DRGs of 3~4 week old SD rats). Since we observed significantly increased TNF-α in rat hindpaw after CFA injection, and mechanical hyperalgesia develops in rats after direct treatment with TNF-α, the pro-inflammatory cytokine TNF-α was applied to mimic the *in vivo *inflammatory condition. TNF-α (5 nM) was added to the culture medium at 3 days *in vitro *(DIV) and maintained for up to 48 hours. As shown in Figure 6A, mRNA levels of TRPV1, BDNF and trkB were differentially enhanced after TNF-α treatment between 6 and 12 hours. In contrast, mRNA levels of CGRP remained unchanged after TNF-α treatment, similar to the results obtained from DRG of hyperalgesic animals (Figure 3A). Similarly, IHC analyses further revealed that TNF-α addition progressively increased BDNF, trkB and CGRP protein in DRG cultures after 24 and 48 hours (Figure 6B - 6J).

**Figure 7:**
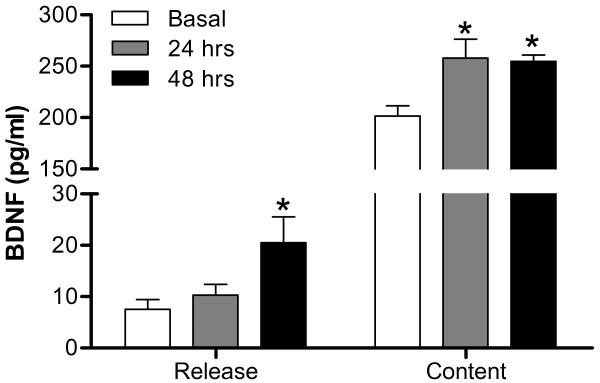
**TNF-α-induced BDNF content and release in DRG cultures**. After a 5 nM TNF-α treatment for 24 and 48 hours, medium and lysed cells were collected and analyzed by BDNF EIA. BDNF release represents basal or TNF-α-induced BDNF in the culture medium; BDNF content means basal or TNF-α-induced BDNF remaining in cultured DRG cells. Data are expressed as mean ± SEM and were analyzed using one-way ANOVA followed by Tukey test, *p < 0.05; compared to corresponding basal groups (N = 5 per group).

To further confirm the impact of TNF-α on BDNF-trkB system in the DRG, TNF-α-induced BDNF release was evaluated by BDNF EIA. After treatment with TNF-α for 24 or 48 hours, BDNF release and expression were analyzed in supernatant medium and cultured cells, respectively. The results show that BDNF release was significantly enhanced after TNF-α treatment for 48 hours (Figure 7). Total BDNF content in DRG cultures was also increased 24 and 48 hours after TNF-α treatment (Figure 7).

### Expression of BDNF-trkB in cultured DRG neurons after neurotrophin treatment

**Figure 8:**
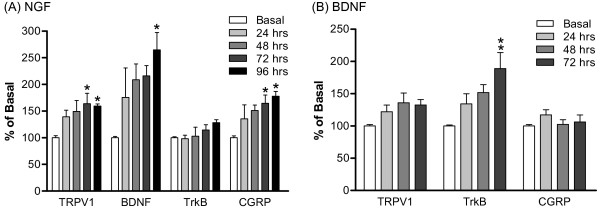
**Expression of TRPV1, BDNF, trkB and CGRP mRNA in DRG cultures after NGF or BDNF treatment**. (A) TRPV1, BDNF, trkB and CGRP mRNA expression after 100 ng/ml NGF treatment, once daily for 24-96 hours. (B) TRPV1, trkB and CGRP mRNA expression after 100 ng/ml BDNF treatment, once daily for 24-72 hours. Data are expressed as mean ± SEM, presented as percentage of basal (no drug treatment) levels, and were analyzed using one-way AVOVA followed by Tukey test. *p < 0.05, **p < 0.01; compared to corresponding basal groups (N = 5~8 per group).

It has been reported that NGF increases the expression of BDNF in peptidergic small neurons in DRG and the release of BDNF was targeted to spinal cord lamina II [17,18]. In order to understand the impact of neurotrophins on inflammation-related proteins, NGF and BDNF (100 ng/ml) were individually applied to DRG cultures for up to 96 hours (Figure 8). Levels of TRPV1, BNDF, trkB and CGRP mRNA were then quantified via real-time PCR.

The results show that NGF induced a significant time-dependent increase in mRNA levels of TRPV1, BDNF and CGRP (Figure 8A). On the other hand, BDNF had no effect on mRNA expression of TRPV1 and CGRP, but significantly up-regulated the mRNA expression of trkB in a time-dependent manner (Figure 8B).

### TrkB receptor-mediated ERK1/2 signaling in DRG cultures after TNF-α treatment

**Figure 9:**
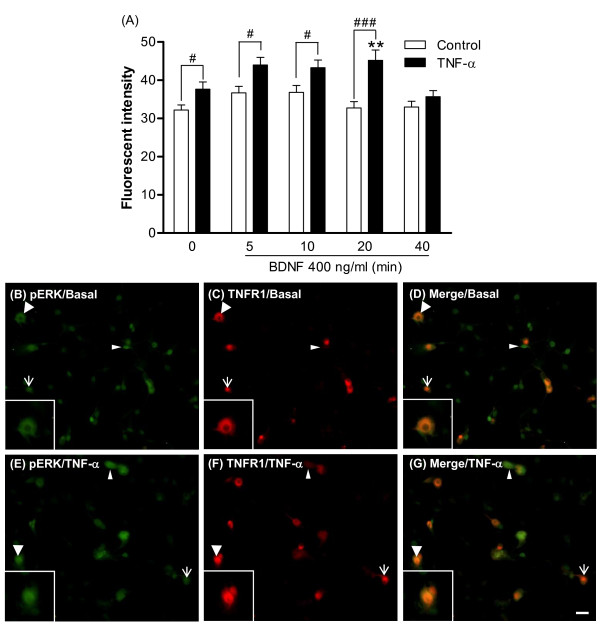
**BDNF-induced phospho-ERK signals and co-localization of phospho-ERK and TNFR1 after TNF-α treatment in DRG cultures**. Phospho-ERK signals were detected after 5 nM TNF-α pre-treatment for 48 hours followed by 400 ng/ml BDNF for 0-40 min. Phospho-ERK and TNFR1 proteins were detected by double immunofluorescent stain. (A) Quantitative measurements of phospho-ERK fluorescent intensity in DRG cultures. Data are presented as fluorescent intensity of immunopositive neurons, expressed as mean ± SEM, and were analyzed using one-way ANOVA followed by Tukey test. **p < 0.01; compared to corresponding controls; ^#^*p *< 0.05, ^###^*p *< 0.001; comparison between TNF-α and time-matched control groups (N = 9 per group). (B-G) After TNF-α treatment for 48 hours, an acute treatment with BDNF was applied for 10 min in cultured DRG neurons. Phospho-ERK is shown by green fluorescence and TNFR1 is shown by red fluorescence. (B-D) Photomicrographs illustrate the immunoreactivity of phospho-ERK (B) and TNFR1 (C) in DRG cultures of the control group (no TNF-α pre-treatment). (D) Merged image of (B) and (C). (E-G) Photomicrographs illustrate the immunoreactivity of phospho-ERK (E) and TNFR1 (F) in DRG cultures of the TNF-α group. (G) Merged image of (E) and (F). Inserts illustrate the phospho-ERK and TNFR1 signals that co-localized in a DRG neuron. Wide arrow, co-localized phospho-ERK and TNFR1; narrow arrow, TNFR1; arrowhead, phospho-ERK. Scale bars, 50 μm.

**Figure 10:**
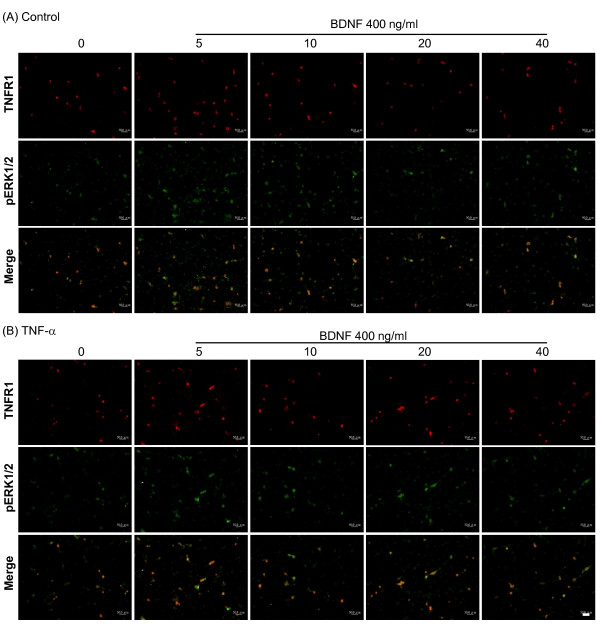
**BDNF-induced phospho-ERK immunohistochemical signals in DRG cultures after TNF-α treatment**. Phospho-ERK signals were detected after a 5 nM TNF-α pre-treatment for 48 hours followed by 400 ng/ml BDNF for 0-40 min. Phospho-ERK and TNFR1 proteins were detected by double immunofluorescent stain. Photomicrographs illustrate the protein signals of individual phospho-ERK and TNFR1 or merged phospho-ERK/TNFR1 in control (no TNF-α pre-treatment) (A) and TNF-α groups (B). Scale bars, 50 μm.

**Figure 11:**
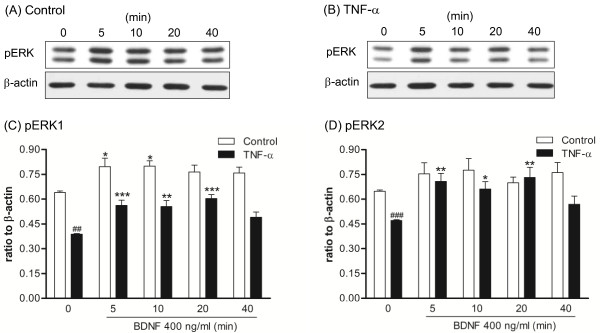
**BDNF-induced phospho-ERK1/2 in DRG cultures after TNF-α treatment**. DRG cultures were treated with 5 nM TNF-α for 48 hours prior to 400 ng/ml BDNF. Protein signals were detected by western immunoblot up to 40 min after BDNF treatment. (A, B) Gel pictures illustrating the phospho-ERK1/2 and β-actin signals in control (no drug treatment) and TNF-α-treated groups, respectively. (C, D) Quantitative measurements of phospho-ERK1 and phospho-ERK2 signals in control and TNF-α-treated groups, respectively. The results are presented as ratios relative to β-actin. The data are expressed as mean ± SEM and were analyzed using one-way ANOVA followed by Tukey test, *p < 0.05, **p < 0.01, ***p < 0.001; compared to time 0. The comparison between basal TNF-α and control groups were analyzed using an un-paired Student's *t-*test; ^##^*p *< 0.01, ^###^*p *< 0.001, (N = 6 per group).

**Figure 12:**
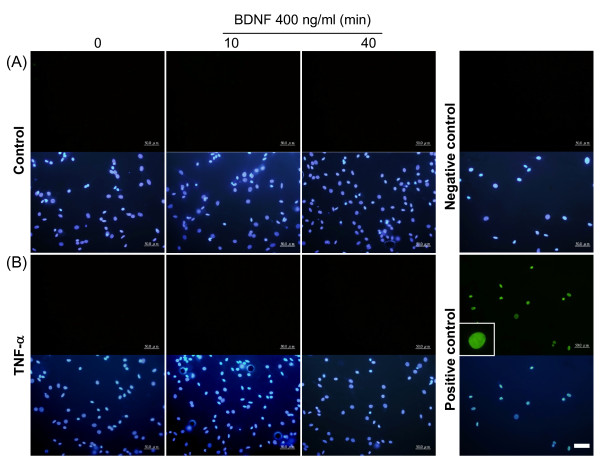
**Apoptosis analyses after acute BDNF treatment after TNF-α treatment**. DRG cultures were pre-treated with 5 nM TNF-α for 48 hours followed by 400 ng/ml BDNF for 10 or 40 min. Cells were immediately fixed and apoptotic cells were detected by TUNEL reaction kit. (A) Control group; (B) TNF-α-pretreated group. DNase I-treated cells served as a positive control, while reaction enzyme removal served as a negative control. TUNEL staining was detected with a fluorescent green dye and cells were counterstained with DAPI. Insert illustrates an apoptotic cell from the positive control. Scale bars, 50 μm.

To test the functional significance of TNF-α-dependent trkB receptor up-regulation, trkB-mediated signaling was evaluated in DRG cultures. BDNF (400 ng/ml) was acutely applied (0, 5, 10, 20, 40 min) to DRG cultures after the cells were treated with 5 nM TNF-α for 48 hours, and then the downstream signals of phospho-ERK were measured by immunefluorescent stain. Further, to determine if TNF receptor co-localized with phospho-ERK signals, double fluorescent stain was employed. The results clearly show that phospho-ERK is enhanced after acute BDNF treatment for 10 min and is further increased by 48 hours of TNF-α pre-treatment (Figure 9B, 9E). Morphological results also reveal that a significant amount of BDNF-evoked phospho-ERK signal is co-expressed with TNFR1, and this occurred mainly in the cytoplasm (Figure 9D, 9G). A complete profile of time-dependent, BDNF-evoked phospho-ERK signal for both control and TNF-α treated groups was evaluated. Quantitative measurements of fluorescent intensity showed that BDNF induced a clear time-dependent enhancement in phospho-ERK signal in the TNF-α pre-treated group, but only a trend toward an increase in the control group (Figure 9A; representative photomicrographs are shown in Figure 10). Because TNF-α pre-treatment elevated basal phospho-ERK, we re-analyzed the data by normalization to corresponding controls, and these results display a similar profile, i.e. BDNF-induced phospho-ERK signals were enhanced in the TNF-α pre-treated group as compared to control (a significant level was reached at 20 min post-BDNF treatment; data not shown). In addition, phospho-ERK1/2 signals were analyzed by western blot of DRG cultures. These results show that BDNF-evoked phospho-ERK1 and phospho-ERK2 signals were both increased time-dependently after TNF-α pre-treatment (up to 20 min) and that this increase is more pronounced than that seen in controls (Figure 11). It is also noticed that basal phospho-ERK1/2 were decreased in the TNF-α pre-treated group in this analysis (Figure 11). No apoptotic cells were identified by TUNEL analysis after 5 nM TNF-α treatment for 48 hours or after acute BDNF administration (400 ng/ml) for 10 or 40 min (Figure 12).

### The effect of TNF-α pre-treatment and/or BDNF on CGRP and substance P release in DRG cultures

**Figure 13:**
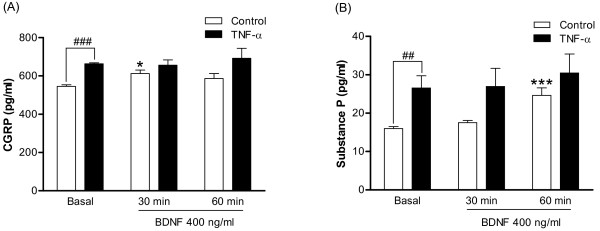
**Basal and drug-evoked CGRP and substance P release in DRG cultures**. After a 5 nM TNF-α treatment for 48 hours, 400 ng/ml BDNF was added for 30 or 60 min. The supernatants were collected and subjected to CGRP and substance P EIA analysis. Data are expressed as mean ± SEM, presented as percentage of control. Data from BDNF treatment were analyzed using one-way AVOVA followed by Tukey test, *p < 0.05, ***p < 0.001; compared corresponding basal groups. The comparison between TNF-α and control groups was analyzed using an un-paired Student's *t*-test, ^##^*p *< 0.01, ^###^*p *< 0.001 (N = 5~7 per group).

To characterize the functional role of TNF-α-enhanced BDNF-trkB signaling, CGRP and substance P were evaluated in DRG cultures. Protein levels of CGRP were significantly enhanced in DRG of either CFA-treated (Figure 3) or TNF-α-treated hyperalgesic rats (Figure 4) and in TNF-α-treated DRG cultures (Figure 6). To assess whether TNF-α can induce CGRP release from cultured DRG, a CGRP EIA was used. After treatment with 5 nM TNF-α for 48 hours, a significant increase in basal CGRP release (approximately 21.6%) was observed (Figure 13A). Moreover, to investigate if TNF-α-enhanced CGRP was functionally associated with up-regulated trkB receptor, BDNF (400 ng/ml) was applied to the culture medium for 30 or 60 min followed by CGRP detection. The results show that BDNF significantly enhanced basal CGRP release after treatment for 30 min (approximately 12.1%) but could not potentiate CGRP release in TNF-α-pre-treated DRG cultures (Figure 13A).

Next, we examined whether BDNF could trigger another nociception mediator, substance P release from DRG, after TNF-α treatment. After treatment with 5 nM TNF-α for 48 hours, an acute treatment with BDNF (400 ng/ml) was applied for 30 or 60 min and the amount of substance P in the culture medium was analyzed using an EIA kit. As shown in Figure 13B, basal substance P release was significantly increased (approximately 65.8%) after TNF-α treatment. Similar to the response of CGRP release, basal substance P release was significantly increased after BDNF stimulation for 60 min. However, BDNF-evoked substance P release did not differ between control and the TNF-α-pre-treated groups.

### Retrograde transport of TNF-α in CFA-induced inflammatory hindpaw

**Figure 14:**
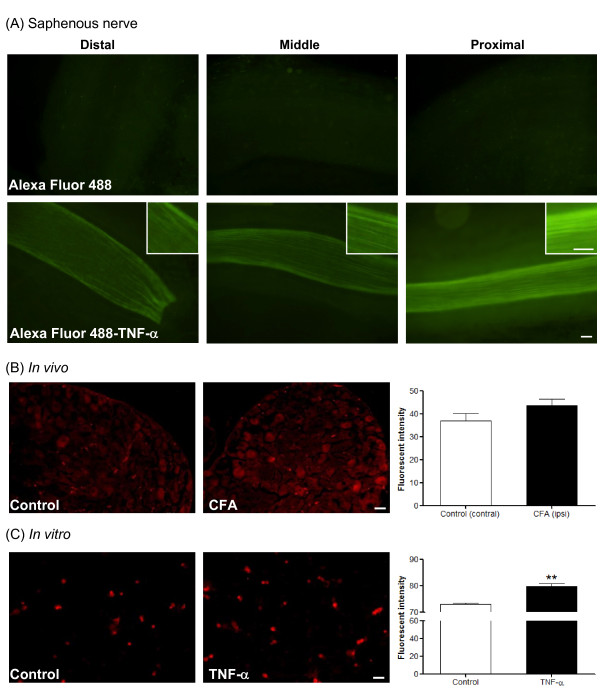
**Retrograde transport of TNF-α and TNFR1 expression in DRG**. (A) After CFA injection (48 hours), TNF-α (labeled with Alexa Fluor 488) was injected into rat hindpaw with free Alexa Fluor 488 as a control. Twenty-four hours later, saphenous nerve was isolated and fluorescent signals were detected using a fluorescence microscope. Photomicrographs illustrate three different segments: Distal, middle and proximal. Scale bars, 50 μm. Inserts illustrate magnified axon fibers labeled with Alexa Fluor 488 with scale bars of 20 μm. (B-C) Photomicrographs illustrate TNFR1 immunoreactivity, detected by immunefluorescent stain. (B) Seventy-two hours after CFA injection, L3-L5 DRGs were collected. (C) TNF-α treatment for 48 hours in DRG cultures. Scale bars, 50 μm. Bar graphs represent quantitative measurements of TNFR1 fluorescent intensity. The data are presented as fluorescent intensity of immunopositive neurons, expressed as mean ± SEM and were analyzed using an un-paired Student's *t-*test. **p < 0.01 (N = 3 per group); compared to control.

Retrograde axonal transport of TNF-α has been reported in various pathological conditions [27,28]. For example, retrograde transport of TNF-α is mediated by TNFR1 in a carrageenan-induced inflammation animal model [27]. Hence, we examined whether TNF-α would undergo retrograde transport from rat hindpaw along the saphenous nerve during CFA-induced inflammation. After CFA injection, Alexa Fluor 488-conjugated TNF-α (30 μl) was injected into rat hindpaw. Saphenous nerve was isolated 24 hours later. The results show green fluorescence in the entire saphenous nerve of the Alexa Fluor 488-TNF-α-treated group, but not in dye-only controls (Figure 14A). Fluorescent intensity increased from distal (0.5 cm from the heel) to proximal (2 cm from the heel). To test if TNF-α transport possibly involves TNFR1, the amount of TNFR1 was measured in DRG sections 72 hours after CFA injection (*in vivo*) and in DRG cultures 48 hours after TNF-α treatment (*in vitro*). As shown in Figure 14B and 14C, the amount of TNFR1 protein was significantly increased in DRG cultures after TNF-α treatment, but we were not able to demonstrate a statistically significant increase after CFA injection.

## Discussion

Numerous studies have attempted to understand the cellular mechanism underlying hyperalgesia and allodynia using a CFA-induced inflammation pain model. Our study focused on the neurochemical changes in lumbar DRG and clearly shows that protein levels of trkB, BDNF and CGRP are significantly increased after animals develop hyperalgesia. Up-regulation of BDNF and CGRP in DRG during inflammation is a well-known phenomenon along with the consequent effect of post-synaptic trkB over-expression in the dorsal horn of spinal cord [18,29]. On the other hand, up-regulation of trkB in DRG during progression of inflammation has rarely been documented. Lee *et al*. (1999) reported that mRNA of truncated trkB receptor increases in DRG after CFA injection, but the full-length trkB receptor does not. Further, Chien *et al*. (2007) reported that BDNF, CGRP, trkA and p75^NTR ^are increased in DRG after CFA treatment [30], while levels of trkB receptor do not change. In addition to these reports, trkB expression and phospho-trkB signal have been found to be enhanced in rat DRG after the insults of colitis or cystitis as well as during spinal cord injury [31-33]. These inconsistent findings imply that expression of DRG trkB receptor during inflammation is variable, possibly due to the complexity of chemical mediators that are recruited in designated disease progression.

In the present study, we found that BDNF protein but not mRNA was significantly enhanced in DRG during inflammation. Previously, it has been reported that levels of BDNF mRNA increase significantly in DRG 24 hours after CFA-injection [29,34], while CFA-induced BDNF protein is up-regulated in DRG (L5) for up to 4 days [35]. Since we observed an increase in the amount of BDNF protein in DRG 72 hours after CFA injection, it is possible that transcriptional regulation occurs only during an early phase while translational regulation lasts longer. In further support of this concept, TNF-α was previously found to enhance BDNF protein expression at 24 hours, but to increase mRNA expression at 4 hours [36]. Consistent with this notion, we did detect an up-regulation of BDNF mRNA 6 hours after TNF-α treatment in DRG cultures. The differential regulation of transcription and translation might also explain the results obtained in CGRP mRNA and protein, both *in vivo *and *in vitro*.

Our results reveal a statistically significant increase in protein levels of trkB, BDNF and CGRP as well as mRNA of trkB and TRPV1, mainly in L3, but CGRP protein in L4 and trkB and CGRP proteins in L5 DRG after CFA treatment. On the other hand, TNF-α treatment led to increased protein levels of trkB, BDNF and CGRP predominantly in L4, with relatively minor effects in L3 and L5 DRGs. This segregated effect was unexpected because rat hindpaw are innervated by primary afferents from L3, L4 and L5 DRGs. A previous report regarding the nociceptive dermatomes in Wistar rat hindpaw demonstrated that the L3 dermatome is contained in the inner part of the paw; the L4 dermatome is contained in the middle part of the paw and the L5 dermatome is contained in the outside part of the paw [37]. Although swelling was visually observed in the entire hindpaw, a differential response was found among the individual segments of lumbar DRG. In support of the notion that segments of the lumbar DRG may respond differently to injury, it has been previously reported, for an animal model of neuropathic pain via L5 spinal nerve ligation, that expression of BDNF and TRPV1 are up-regulated in uninjured L4 DRG [38]. Similarly, expression of TNFR1 and TNFR2 are increased in injured L5 and uninjured L4 DRG after L5 spinal nerve ligation [39,40]. In addition, our results showing differential effects in L3 and L4 in response to CFA and TNF-α might be due to drug formulation, i.e. chyle form of CFA vs. liquor form of TNF-α. A detailed explanation of the cause of uneven sensitivity between L3, L4 and L5 DRGs after CFA or TNF-α hindpaw treatment requires further investigation.

Pro-inflammatory cytokines play an important role in the process of inflammatory pain and their release results in pain sensitization [2,3]. TNF-α is a potent cytokine, and intraplantar injection of TNF-α in rats has been previously shown to produce mechanical hyperalgesia while TNF-neutralizing antibodies reverse TNF-α-induced nociception [1,8]. Furthermore, TNF-α increases expression of TRPV1 in DRG via TNFR1 receptor with a requirement for ERK signaling [11]. Interestingly, anti-TNF-α treatment in patients with rheumatoid arthritis results in decreased plasma BDNF [41]. TNF-α can also enhance expression of BDNF protein in trigeminal ganglion neuronal cultures [36]. Hence, it is plausible that a casual relationship between TNF-α and the BDNF-trkB system in DRG mediates inflammation-induced hyperalgesia. In this study, we first demonstrated that levels of TNF-α were raised significantly in the inflamed tissues, as compared to uninjured hindpaw, thereby confirming a role for TNF-α during inflammation. Further, we proved that inflammatory hyperalgesia could be induced by direct TNF-α injection into hindpaw. The finding that TNF-α in DRG is undetectable after animals develop hyperalgesia is surprising, but may be explained by the following considerations. First, mono-arthritis developed under the current experimental conditions, which amounts to an early inflammatory stage confined mainly to the affected tissues. The resulting inability to detect TNF-α in serum is similar to the findings of a previous report [42], and seems to support this argument. Second, TNF-α produced locally in injured tissues would act on TNF-α receptors found on free nerve endings and undergo retrograde transport to DRG. This phenomenon was recently demonstrated in a rat model of carrageenan-induced inflammation [27] as well as a rat model of peripheral nerve injury [28]. In the present study, we validated the ability of TNF-α to undergo retrograde transport along the saphenous nerve in a CFA-induced inflammatory rat model. The axonal transport pattern and speed are comparable with previous observations using a carrageenan-induced pain model [27]. Hence, rather than massive TNF-α accumulation in DRG that would be expected to occur at a late stage of inflammation (progressive poly-arthritis), we speculate that in the current model, intracellular TNF-α carried by the TNF-α receptor is the main participant in the DRG, although we could not exclude the possibility that TNFR1 expression resulted from inflammation while independent from translocation.

Similar to the CFA-induced inflammatory condition, we found that TNF-α injection into hindpaw and application to DRG cultures produced a clear enhancement of trkB and BDNF expression in DRG. In addition, we observed a release of BDNF from DRG cultures after TNF-α treatment. Other than targeting the post-synaptic trkB receptor in the dorsal horn, we speculate that enhanced BDNF would also have chance to activate local trkB receptors in DRG, leading to trkB dimerization and auto-phosphorylation, which would ultimately trigger pre-synaptic signaling pathways [43]. In support of this hypothesis, our results demonstrate that activation of trkB receptors by BDNF enhances phospho-ERK1/2 signal in TNF-α-pretreated animals, suggesting that a possible intra-DRG BDNF-trkB system could be functionally self-activated under chronic TNF-α exposure. However, we observed an inconsistent result for basal phospho-ERK1/2 signal after TNF-α pretreatment (i.e. an increase in IHC but a decrease in western blot). Other than a difference possibly due to analytical methods, the impact of TNF-α on overall basal MAPK signal requires further investigation. The dual observations that both BDNF and BDNF-triggered phospho-ERK signals largely co-localize with TNFR1 suggest that TNFR1 might associate with *in situ *BDNF-trkB up-regulation. In addition to the effect of TNF-α on BDNF-trkB, it is well known that TNF-α can induce IL-6, IL-8 and granulocyte-macrophage colony-stimulating factor (GM-CSF) secretion through activation of MEK1/2, p38 MAPK and NF-κB pathways during inflammation [44,45]. Since a notable inflammation was observed in both CFA- and TNF-α-treated hindpaw, we could not exclude the possibility that a combined action of these chemical mediator(s) might work synergistically to up-regulate the BDNF-trkB system *in vivo*.

NGF is an important mediator of inflammatory pain [13]. After release from immune cells, NGF binds with trkA to form an NGF/trkA complex which is then retrogradely transported to DRG to up-regulate expression of genes, such as those for TRPV1, BDNF, CGRP and substance P [2,3] via ERK, PI3K, p38 MAPK or CaMKII signaling [5,14,15,46]. TNF-α has also been shown to enhance NGF expression *in vivo *[42], although NGF does not appear to be involved in TNF-α-mediated BDNF up-regulation in trigeminal ganglia cultures [36]. Our observation that TNF-α and NGF can both induce BDNF expression in DRG cultures suggests a functional synergism between these two chemical mediators. However, the increase of BDNF by TNF-α may be derived from astrocytes within the cultures [47]. Although Ara-C was applied in our DRG culture system, we cannot totally exclude the possibility that enhanced TNF-α/NGF-induced BDNF could be partially due to contaminating satellite cells.

Our results show that BDNF treatment increases mRNA expression for trkB receptor in DRG cultures. BDNF is reported to co-express with trkA (20%) and trkB (10%) receptors in DRG [18,21]. In addition, BDNF is thought to act as an autocrine or paracrine signal regulating trkB in sensory neurons [16,48]. Low doses of BDNF up-regulate trkB receptors while high doses down-regulate trkB receptors [49]. Our *in vitro *results demonstrate that an increase in trkB receptors is triggered by either BDNF or TNF-α. This effect may eventually form a sensitization loop to increase pain transmission at the level of the DRG.

The finding that basal CGRP release is enhanced in DRG after chronic TNF-α treatment supports a previous study which showed that acute TNF-α treatment increases CGRP release in rat trigeminal ganglia [50]. In addition, these results also correspond with our *in vivo *and *in vitro *observations that total CGRP protein increases after CFA-induced inflammation [36,50]. Similarly, we found that chronic TNF-α treatment results in enhanced basal substance P release in DRG cultures. Acute BDNF stimulation could induce an increase in substance P release 60 min after treatment, but had no effect after TNF-α pretreatment. The findings are consistent with previous reports [23,31] that BDNF can stimulate CGRP and substance P release under basal conditions via trkB receptor in spinal cord. TNF-α regulation of substance P release has never been studied in DRG, though Ansal *et al *[51] reported that substance P induces TNF-α mRNA expression and release in mast cells. In addition, substance P enhances mechanical allodynia and heat hyperalgesia in pre-protachykinin A^-/- ^mice through the actions of NGF and TNF-α [52]. In this manuscript, we demonstrate that TNF-α pre-treatment enhances the production and basal release of CGRP and substance P, consequently facilitating pain transmission. The lack of a trkB effect on CGRP/substance P release during inflammation suggests that a maximal effect is already achieved by TNF-α pre-treatment.

## Conclusions

**Figure 15:**
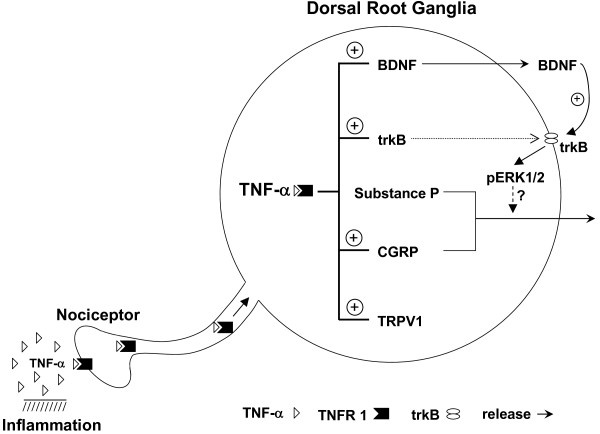
**Working model of BDNF-trkB up-regulation in DRG during TNF-α-mediated inflammation and hyperalgesia**. Upon inflammation, TNF-α may be released near nociceptors where it binds TNFR1 and undergoes retrograde transport to DRG via TNFR1. In the DRG, TNF-α enhances expression of BDNF, trkB, CGRP and TRPV1, through either transcriptional or translational regulation. The enhanced BDNF is released locally to act as an autocrine or paracrine signal on pre-synaptic trkB receptors in the DRG. Consequently, activated trkB receptor increases pain mediators, CGRP and/or substance P release and facilitates pain transmission, possibly via phospho-ERK1/2.

We hypothesize that during chronic inflammation, cytokines such as TNF-α are released locally and act on TNF receptors in nociceptors. TNF-α then undergoes retrograde transport to the DRG to enhance synthesis of BDNF, trkB and CGRP. NGF may also be released from immune cells around nociceptors and bind to trkA receptor to further increase the expression of TRPV1, BDNF and CGRP. The enhanced BDNF in DRG might have chance to act as an autocrine and/or paracrine signal to activate pre-synaptic trkB receptors synergistically with cellular events mediated by TNF-α (or by other inflammatory mediators), and up-regulate synaptic excitability in pain transmission (see Figure 15 for a working model). The identification of the cellular mechanism(s) underlying the DRG BDNF-trkB system in inflammatory pain may provide novel therapeutic targets to combat chronic pain.

## Competing interests

The authors declare that they have no competing interests in conducting this study.

## Authors' contributions

YTL carried out all the *in vivo *and *in vitro *experiments in this study and prepared the first draft of this manuscript. JCC conceived of the study, participated in its design and coordination and helped to draft the manuscript. LSR and HLW participated in experimental design and consulted on the study. All authors read and approved the final manuscript.
